# Building the Digital Mental Health Ecosystem: Opportunities and Challenges for Mobile Health Innovators

**DOI:** 10.2196/27507

**Published:** 2021-10-13

**Authors:** Benedetta Spadaro, Nayra A Martin-Key, Sabine Bahn

**Affiliations:** 1 Cambridge Centre for Neuropsychiatric Research Department of Chemical Engineering and Biotechnology University of Cambridge Cambridge United Kingdom; 2 Psyomics Ltd Cambridge United Kingdom

**Keywords:** digital implementation, digital mental health, digital psychiatry, digital technology, viewpoint

## Abstract

Digital mental health technologies such as mobile health (mHealth) tools can offer innovative ways to help develop and facilitate mental health care provision, with the COVID-19 pandemic acting as a pivot point for digital health implementation. This viewpoint offers an overview of the opportunities and challenges mHealth innovators must navigate to create an integrated digital ecosystem for mental health care moving forward. Opportunities exist for innovators to develop tools that can collect a vast range of active and passive patient and transdiagnostic symptom data. Moving away from a symptom-count approach to a transdiagnostic view of psychopathology has the potential to facilitate early and accurate diagnosis, and can further enable personalized treatment strategies. However, the uptake of these technologies critically depends on the perceived relevance and engagement of end users. To this end, behavior theories and codesigning approaches offer opportunities to identify behavioral drivers and address barriers to uptake, while ensuring that products meet users’ needs and preferences. The agenda for innovators should also include building strong evidence-based cases for digital mental health, moving away from a one-size-fits-all well-being approach to embrace the development of comprehensive digital diagnostics and validated digital tools. In particular, innovators have the opportunity to make their clinical evaluations more insightful by assessing effectiveness and feasibility in the intended context of use. Finally, innovators should adhere to standardized evaluation frameworks introduced by regulators and health care providers, as this can facilitate transparency and guide health care professionals toward clinically safe and effective technologies. By laying these foundations, digital services can become integrated into clinical practice, thus facilitating deeper technology-enabled changes.

## Introduction

Mental health disorders represent the leading cause of disability worldwide, with over one third of the world’s population being affected by a mental health condition in their lifetime [1]. Given the increasing pressures on mental health care budgets globally, as well as shortages of health care professionals who are facing an overwhelming growing burden of chronic and recurring mental health conditions, prevention strategies and improvements in early identification and treatment are essential. To this end, mobile health (mHealth) tools such as apps can offer innovative ways to help develop and facilitate mental health care provision. Indeed, the unmet need for psychiatric services has sparked significant interest in developing apps to meet patient demand. As a result, more than 10,000 apps addressing mental health symptoms such as anxiety, low mood, and insomnia are now available on the Apple App Store and Google Play Store [2]. Furthermore, despite its catastrophic effects, the COVID-19 pandemic has been a pivot point for digital health implementation, with some describing it as years’ worth of digital transformation in just a few months [3-5]. This transformation has been made possible by the recognition from governments and regulatory bodies of the need for speed, flexibility, and action in response to the pandemic [6-8]. However, many current mHealth technologies rely heavily on patients’ ability to self-diagnose and use self-help rather than being fully integrated into the clinic. In building a holistic and comprehensive digital mental health ecosystem that *augments* rather than *replaces* traditional mental health care approaches, strategies for the design, validation, and implementation of mHealth technologies should be carefully considered by innovators moving forward.

## mHealth: Promise of Dimensional Psychiatry and Digital Phenotyping

Psychiatric nosology, as described in gold-standard diagnostic manuals, has often been criticized for being overly narrow, failing to capture the wide range of clinical symptoms that are endorsed by individuals with mental health conditions [9,10]. In this regard, moving away from a categorical, symptom-count approach to a transdiagnostic view of psychopathology may facilitate early and accurate diagnosis, and allow for personalized treatment strategies. Given that time is a premium in clinical settings, where relying on brief symptom-count checklists such as the Patient Health Questionnaire-9 [11] and the seven-item Generalized Anxiety Disorder questionnaire [12] is common practice, digital technologies may offer an innovative way to improve diagnostic accuracy and advance mental health care provisions. Indeed, digital technologies such as mHealth apps have the capacity for the collection of a vast range of key transdiagnostic data via active reporting (ie, self-administered symptom reporting or monitoring via an app), providing valuable information on relevant social and demographic factors as well as current and past symptom profiles [13]. Digital technologies, including adaptive/nonlinear questionnaires where patients are required to answer questions based on previous answers, can further personalize and streamline the collection of active cross-disorder symptom data.

Moving beyond active symptom data, passive data, which measure aspects of daily living and can be collected continuously in the background, such as activity rhythms, text and call logs, sleep quality, keyboard reaction time [14], speech phonation, coherence, sentiment, and language patterns [15], can be applied to better inform mental health assessments in a process called digital phenotyping [16]. Data platforms that gather information from various digital sources have started emerging with the goal of collecting large-scale epidemiological and longitudinal data to identify risk factors and invest in mental health resources effectively. An example of this approach is offered by the recently launched Mental Health America (MHA) dashboard supported by MHA and Lundbeck. The dashboard collects publicly available data and anonymized data from web screening tools offered by MHA to highlight mental health hotspots across the United States. The objective is to implement advanced targeted interventions, address disparities in a timely manner, and design policies for at-risk populations [17,18]. Furthermore, advances in mHealth platforms (eg, Apple HealthKit or Google Fit) also allow for the bundling of data from different sensors such as accelerometers, microphones, GPS sensors, and gyroscopes, enabling the collection of physical and mental health information [19].

Critically, digital phenotyping for mental health is still in its infancy, with further research into how these data fit into or build upon the current diagnostic guidelines and criteria being a necessity moving forward. Both active as well as passive data collection also raise potential serious ethical concerns, which will need to be legislated and regulated. In the future, combining data from active and passive monitoring sources may feed new data into mental state examinations, allowing for more comprehensive data collection outside the time-pressured environment of clinical consultations. In turn, mHealth technologies have the potential to streamline mental health care delivery and management by reducing the number of in-person appointments, thereby freeing up clinician time, resulting in cost savings for both the provider and patient. However, these technologies usually require patients and clinicians to engage in new behaviors, which may be accompanied by reluctance to change. As a result, uptake of mHealth tools critically depends on perceived relevance, effectiveness, confidentiality, and engagement of the end users. A way to address this is by utilizing behavior theories and engaging in codesigning approaches when designing digital mental health care solutions.

## Designing mHealth Tools With Patients and Clinicians in Mind

Understanding the target patient population’s need is pivotal to the design of effective mHealth tools. To this end, behavior theories such as the Health Belief Model (HBM) [20], Behavioral Intervention Technology (BIT) model [21], and behavior model for Just-in-Time Adaptive Interventions (JITAI) [22] offer frameworks that help identify key drivers and barriers to the uptake of new technology, starting from app subscription to adherence to recommendations delivered by mHealth tools [23-25]. For instance, the HBM can help mHealth developers better understand their target patient population by investigating their inclination to seeking mental health support and what drives their adherence to treatment (eg, trust in clinicians, severity of illness, social stigma). The HBM is based on the theory that a person’s willingness to engage in treatment is primarily due to their health perceptions. Indeed, mental health studies have shown that the perceived severity of mental illnesses impacts treatment outcomes (eg, adherence and beliefs about treatments) [26]. Thus, using the HBM can inform developers on how to best engage patients in adhering to the use of a novel digital tool.

Similarly, the BIT model combines the understanding of technological features of mHealth tools with behavioral principles to guide innovators on how to design and implement technology that is useful and usable. For instance, motivational enhancement strategies (behavior principle) can be used in the form of push notifications (technological feature) to prompt patients to track their mood regularly. The BIT model approach can be especially valuable for researchers looking to develop multifunctional tools for mental health, including tools for mood monitoring and treatment such as electronic cognitive behavioral therapy, as well as tracking adherence to medication. In these cases, the stepwise, structured approach of the BIT model can best inform developers on how to use different behavioral principles and technologies to achieve their clinical aims.

The JITAI model can be employed by researchers developing adaptive sampling and ecological momentary assessments aimed at collecting information at sufficient frequencies and quantities to be useful without overburdening mental health patients. Critically, a delicate balance between utility and tolerability must be sought when developing mHealth monitoring tools in mental health, especially in conditions with specific triggers (eg, social anxiety triggered by meeting strangers, harmful alcohol use triggered by stress). The behavioral model for JITAI offers a framework to systematically identify when patients are most vulnerable to experiencing symptoms and pinpoints circumstances in which patients may be most receptive to the digital intervention. 

Transitioning into the digital mental health care space will also involve cultivating a collaborative approach that includes academics, software developers, computer and data scientists, investors, and health care professionals. Indeed, moving forward, the concept of codesigning will need to be at the forefront of development, involving *all* potential users and stakeholders as active collaborators in the creation of digital diagnostics and interventions. This is especially critical for mental health technologies, which require clinical support from health care professionals to encourage engagement. Moving from the view of designing a digital product to designing a technology-enabled service means that the aims, role of the practitioner, and technology are designed and evaluated simultaneously in the relevant context [27]. Shifting from the practice of designing *for* to designing *with* users can help humanize digital health care, and *augment* rather than *replace* traditional health care delivery and management systems. By laying these foundations, technology-enabled services can become a part of everyday clinical practice and set the scene for deeper technology-enabled changes. Critically, as well as leveraging on behavior theories and codesigning approaches to increase engagement and relevance, mHealth developers should also pursue implementation studies to validate and assess the efficacy of their interventions.

## Clinical Trials to Build a Strong Evidence-Based Case for Psychiatric mHealth Tools

Recent studies show that the most commonly downloaded mental health apps mainly fall under the umbrella of well-being apps offering relaxation, meditation, or mindfulness skills rather than validated treatments, and that the scientific language used to support claims often lacks corresponding evidence in the literature [28,29]. Although these apps can be incredibly useful and helpful for some patients, they may not be effective for more severe mental health concerns. Furthermore, many of these mHealth tools rely heavily on patients’ ability to self-diagnose and use self-help rather than being fully integrated into clinical service delivery. In building a holistic and comprehensive digital mental health ecosystem, innovators are encouraged to design technology in a more sophisticated manner, moving away from a one-size-fits-all well-being approach to embrace the development of clinically validated and integrated treatments.

Clinical trials and observational studies can help mHealth developers build a strong clinical case for their technologies and differentiate them from digital wellness apps. Moving forward, it should be noted that randomized controlled clinical trials typically bear little resemblance to clinical settings. Often, recruitment favors clinicians and patients who are interested in using digital health technologies and are therefore more likely to adhere to them. However, these participants may represent only a small proportion of potential users. It should then come as no surprise if digital technologies fail in general health care settings. Thus, innovators should carefully consider the opportunity to conduct the evaluation of digital mental health technologies within the intended context, and to assess both its effectiveness and implementation requirements [27]. This so-called hybrid pragmatic trial design can assess both treatment effects and implementation strategies [30] in real-world patient populations with outcome measurements routinely used in clinical practice [31]. Thus, implementation and sustainment strategies become integral to the clinical evaluation process and part of the intervention design from the very beginning. Importantly, performing clinical studies and citing published literature in the descriptions of digital tools can only partially address the difficulties encountered by providers, payors, and patients in assessing and choosing effective digital interventions. Standardized evaluation frameworks introduced by regulators and health care providers can create transparency and guide health care professionals toward clinically meaningful technologies, with efforts being made in this direction.

## Defining Appraisal Frameworks for mHealth Solutions

Several academic groups have contributed to the design of evaluation frameworks. For instance, the American Psychiatric Association (APA) has developed an app evaluation framework [32] that has been used by the New York Department of Health in the construction of an app library. Following the APA model, Lagan et al [33] designed a health app evaluation model, which harmonized 45 preexisting frameworks and where answers can be either binary or numeric to permit an objective evaluation of (1) background and access, (2) data safety and privacy, (3) app effectiveness and clinical foundation, (4) user engagement, and (5) data integration. The app evaluation model can be used by clinicians and patients to inform decisions on the suitability of candidate apps for the intended clinical use. Mental health apps often require patients to disclose sensitive personal information; thus, data safety and privacy are fundamental. To this end, the framework assesses apps on the possibility of deleting data, the format in which data are shared (nonanonymized, deidentified, anonymized), and whether data are shared with third parties. Thus, data handling procedures should be clearly stated in an app’s description or privacy policy. Ultimately, compliance with data security statutes and regulations (eg, Health Insurance Portability and Accountability Act and General Data Protection Regulation) is paramount for mental health apps collecting sensitive patient data. Clinicians will only recommend apps that fulfill data security requirements and that can easily be integrated into existing clinical workflows.

Regulators and health care providers are introducing additional ways of evaluating and certifying digital health technologies that do not fall under hardware medical device regulations. Innovators should work with regulatory bodies and be aware of the innovation plans for appraisal frameworks. The Food and Drug Administration (FDA) developed the Digital Health Innovation Action Plan to provide a more streamlined and efficient regulatory oversight of digital health technologies [34]. The Plan includes the Software Precertification Pilot Program, which precertifies organizations, not individual products [35]. An “excellence appraisal” is conducted to give the FDA the assurance that the organization is capable of producing high-quality, safe, and effective medical software products so that individual product reviews are carried out more rapidly and swiftly. An international approach to one-off regulatory appraisals also emerged with the Medical Device Single Audit Program (MDSAP), piloted in the United States, Australia, Brazil, Canada, and Japan. The objective of the MDSAP is to develop a single regulatory audit process that can satisfy the needs of multiple regulatory jurisdictions [36]. Europe stands as an observer in the MDSAP, and the new Medical Device Regulation mainly focuses on hardware devices; however, steps have been taken toward a more software-specific approach [37]. In the United Kingdom, the National Health Service (NHS) X developed the beta version of Digital Technology Assessment Criteria for health and social care, which outlines baseline assessment criteria to validate the suitability and function of digital health technologies for use by the NHS, social care staff, or directly by citizens [38]. Trade associations such as the Digital Therapeutics Alliance (DTA) are promoting industry standards and evaluation frameworks to enhance the understanding, adoption, integration, and reimbursement of clinically evaluated digital therapeutics into mainstream health care. The DTA’s member companies include large pharmaceutical companies such as Novartis and Otsuka, as well as digital therapeutics developers such as Pear Therapeutics and SilverCloud, along with technology companies such as Philips, demonstrating the interdisciplinary efforts in pushing forward digital health technologies.

Compliance with data security regulations, assessment against commonly used frameworks, and certifications issued by regulatory bodies can help innovators build transparency and trust with their stakeholders. Importantly, presenting efficacy data and privacy matters in clear terms is essential to engage providers in the uptake of novel digital tools. In other areas of medicine, a clear evidence base, ease of use, and swift integration into clinical workflows have proven to facilitate the uptake of digital health technologies [39,40]. Experts have suggested that the introduction of novel technologies in health care could be facilitated by a dedicated health care profession called a “digital navigator,” whose task in hospitals and clinics would be to select evidence-based apps, troubleshoot, and interpret digital data outputs in a clinically meaningful way [41]. However, mHealth developers should not heavily rely on the presence of specialized personnel; rather, they should focus on communicating evidence transparently, designing tools that fit into current care pathways and information technology system provisions, and providing actionable patient data. Consequently, the use of digital tools will not be limited to settings that have specialized staff members, and as uptake of these apps increases, it will become cost-effective to train and employ digital navigators to support further, more complex implementation of digital health tools.

## Conclusion

Digital mental health technologies can enable the early identification of conditions, and facilitate disease management and treatment while empowering patients to make better informed decisions about their own health. Critically, there has been a movement toward technology that replaces the human element, rather than complementing and enhancing the invaluable involvement of health professionals in patient care. However, it is important not to throw decades or even centuries of clinical knowledge overboard. The way forward to building a digital mental health ecosystem must be more nuanced and sensitive. By developing patient-centric solutions, we can enable earlier and more accurate diagnoses, and in turn connect people with the most appropriate and effective treatment and care. As we move toward a postpandemic world, building the digital mental health ecosystem will require optimizing and adapting traditional mental health care services with considered step-wise innovation approaches, allowing health care professionals and patients to adapt to change. Innovative solutions will not come from leaps forward in technology but rather from new models of codesign, careful use of behavior theories, new pragmatic approaches to clinical evaluation, unprecedented collaboration in defining industry standards, and an open mind to trial-and-error implementation approaches.

The long-term goal for digital mental health technology innovators should be to design tools and services that can easily become so engrained in the health care ecosystem that what is now referred to as *digital health* will simply be called *health*.

## Abbreviations

APA: American Psychiatric Association
BIT: Behavioral Intervention Technology model
DTA: Digital Therapeutics Alliance
FDA: Food and Drug Administration
HBM: Health Belief Model
JITAI: Just-in-Time Adaptive Interventions
MDSAP: Medical Device Single Audit Program
MHA: Mental Health America
mHealth: mobile health
NHS: National Health Service
